# Detection and phylogenetic analysis of kinetoplast DNA of *Leishmania infantum* infected humans, domestic dogs and sandflies in Northwest Iran

**DOI:** 10.1371/journal.pone.0296777

**Published:** 2024-03-13

**Authors:** Hamed Behniafar, Niloofar Taghipour, Adel Spotin, Zabih Zare, Seyyed Javad Seyyed Tabaei, Elham Kazemirad, Vahideh Moin Vaziri, Mehdi Mohebali

**Affiliations:** 1 Department of Medical Parasitology, Sarab Faculty of Medical Sciences, Sarab, Iran; 2 Department of Medical Parasitology and Mycology, School of Medicine, Shahid Beheshti University of Medical Sciences, Tehran, Iran; 3 Department of Tissue Engineering and Applied Cell Sciences, School of Advanced Technologies in Medicine, Shahid Beheshti University of Medical Sciences, Tehran, Iran; 4 Department of Parasitology and Mycology, Faculty of Medicine, Tabriz University of Medical Sciences, Tabriz, Iran; 5 Department of Medical Parasitology & Mycology, School of Public Health, Tehran University of Medical Sciences, Tehran, Iran; 6 Research Center for Endemic Parasites of Iran, Tehran University of Medical Sciences, Tehran, Iran; Iran University of Medical Sciences, ISLAMIC REPUBLIC OF IRAN

## Abstract

Leishmaniasis refers to a disease with a wide range of manifestations; and there are three main forms of disease, cutaneous, mucocutaneous, and visceral. Leishmaniasis is one of the diseases with a protozoan agent which is vector-borne. Visceral leishmaniasis (VL) is the most severe form that can be fiercely life-threatening if left untreated. VL can be caused by members of *Leishmania donovani* complex, in Iran, *Leishmania infantum* is considered the primary causative agent of VL, resulting in a zoonotic form of VL. The two main goals of our work, which followed our prior sero-epidemiological and entomological survey, were to characterize and conduct a phylogenetic analysis of the *Leishmania* species that infect people, dogs, and sandflies. The samples were collected throughout 2017, from January to December, so blood samples were collected from humans and dogs, while sandfly samples were collected with sticky traps. DNA extracted from all seropositive samples of humans and dogs, 10% of sero-negative human samples, and all collected sandflies were subjected to *kDNA*-nested-PCR for tracing parasites. A total of 30 samples, including 20 human samples, 8 dog samples, and 2 sandfly samples, were found positive for the *kDNA* gene of *L*. *infantum*. Sequences were evaluated to study the genetic diversity among the six discovered *L*. *infantum*. Based on *kDNA*, the phylogenetic study of *L*. *infantum* demonstrated a high level of genetic variety and a relationship between the host, the parasite’s geographic origin, and its genetic diversity.

## Introduction

Leishmaniasis is one of the most important zoonotic protozoan diseases in tropical and subtropical countries, including Iran. The disease is caused by the members of the genus *Leishmania* and transmitted by Phlebotominae sandflies. There is a wide range of manifestations from subclinical infection to fatal form; however, cutaneous leishmaniasis (CL), mucocutaneous leishmaniasis, and visceral leishmaniasis (VL) are considered three primary clinical forms of this disease, but only VL and CL are endemic in some parts of Iran [1–3]. A total of 208 357 and 12 383 new cases of CL and VL, respectively, were reported to the WHO in 2020 when the illness was endemic in 98 nations [4]. Although CL is the more common type, VL is important because it may be lethal if neglected [5]. Although VL was sporadically found across Iran, there are two primary foci: Azerbaijan region (East Azerbaijan and Ardabil provinces) and Fars province [2]. For a long time, several counties of East Azerbaijan province have been considered as VL foci, but Kaleybar is one of the most important ones [2].

*L*. *infantum* and *L*. *donovani* cause the fatal form of leishmaniasis in the Old World and *L*. *chagasi* in the endemic areas of the New World. However, in the Mediterranean region, including Iran, VL is caused by *L*. *infantum* [2, 6]. In order to effectively manage the illness, it is essential to understand the epidemiological situation, the species that cause the disease, and the main vector species and animal reservoirs in each location. Providing a safe, convenient, and low-risk diagnostic method for VL is a big challenge, but various studies have been conducted to solve this problem [7, 8]. Direct agglutination test (DAT) is one of the most advised serological methods for diagnosing human and canine VL, and it is widely used [9, 10]. Besides serological methods, we need another tool for species identification of *Leishmania* parasites, which is highly required to anticipate the prognosis, choose proper treatment, and investigate the genetic diversity of isolated parasites [11]. For the abovementioned purposes, molecular techniques are among the best options [12, 13]. Several nuclear DNA markers, including cytochrome b (*CytB*), the internal transcribed spacer (*ITS*), glycoprotein 63 (*gp63*), and kinetoplast DNA (*kDNA*), have been used to identify species and study genetic diversity in *Leishmania* [14–17]. The last one was reported as the most sensitive option to detect the parasite DNA in low parasite load samples [18].

Primary parts of the present study were published, which revealed the seroprevalence of 2.18% and 9.9% in humans and dogs, respectively, and the predominance of Phlebotominae sandflies belonging to Major group *(Larroussius* subgenus) in Kaleybar and Khoda-Afarin counties [19]. Therefore, two main objectives are aimed: 1) Characterizing of *Leishmania* species in the human, dog, and sandfly samples from our previous study using *kDNA* [19]. 2) To perform phylogenetic analysis on PCR-positive samples. To achieve this, samples taken from people, dogs, and sandflies underwent molecular analysis.

## Material and methods

### Ethics

Ethical approval was obtained from two centers, including the committee on the Committee on the Ethics of Research department, School of Medicine, Shahid Beheshti University of Medical Sciences, and Tehran University of Medical Sciences, under permit number: IR.SBMU.MSP.REC.1395.204 and IR-TUMS. 95-01-162-31832. Previous to the study’s enrolment of children and dogs, the research methodology was thoroughly explained, and documented consent was obtained from the parents and owners of the animals.

All procedures performed in this study involving animals were based on the ethical standards of the Animal Ethics Committee of Shahid Beheshti University of Medical Sciences (Approval No.: IR.SBMU.MSP.REC.1395.204). All efforts were made to minimize animal suffering during our study.

Adult participants, the parents of children participating in the research, and the owners of the animals all provided written permission after receiving thorough descriptions of the study.

### Sampling sites

This study was conducted in Kaleybar and Khoda-Afarin counties (latitude of 38° 47’ N and longitude of 46° 58’), East Azerbaijan province, Northwest Iran, in an area covering 3597 km2, which is about 1 240 meters above sea level. The desired area is characterized by a pleasant mountainous climate with an annual temperature of 13.6 degrees Celsius and an average annual precipitation of 372 millimeters. The target counties had 79 120 residents as of the 2016 census, and most lived in rural areas. Animal husbandry is this area’s main economic activity, leading to more significant interactions between dogs and people. Villages were selected for sample collection using the simple random selection method.

### Sample selection for molecular examination

We studied three primary populations within the designated study areas: dogs, humans (specifically children under 12), and sandflies. Human and animal samples were obtained with the explicit consent of the parents/legal guardians of the children and the owners of the dogs. Based on the observed prevalence rates of 2.9%, 15%, and 5% for VL in humans, dogs, and sandflies, respectively, within the region, the sample size formula was utilized to calculate the required sample sizes of 1081, 221, and 477 correspondingly [2, 20]. Subsequently, 1420 human samples, 101 dog samples (due to some restrictions), and 577 sandfly samples were meticulously collected for analysis. All samples are from our previous research, and detailed sampling techniques, sample preparation, serological testing, and sandfly species identification have been described in that [19]. A total of 1 420 human samples (under the age of 12), 101 domestic dog (*Canis familiaris*) samples, and 577 female sandfly samples were collected. All samples were collected from January to December 2017. Seropositive using direct agglutination test (DAT) (titers ≥1:800 for humans and ≥1:80 for domestic dogs), 10% of seronegative blood samples and all collected sandflies were subjected to molecular examinations.

### DNA extraction and PCR amplification

#### DNA extraction from human and dog blood samples and Phlebotominae sandflies

The whole blood samples were washed to eliminate potentially interfering hemoglobin before DNA extraction. Then, DNA was extracted using the phenol-chloroform method and DNA-plus extract kit (Cinagen, Iran). The latter method was performed based on the manufacturer’s instructions and only for a limited number of blood samples. In case of Phlebotominae sandflies, while the head and terminal segments of the abdomen were detached and mounted for species identification, the remainder of the body was preserved at minus 21 degrees Celsius in a 1.5 mL microtube with the same code. Sandfly samples were homogenized before DNA extraction. Then, DNA was extracted using the phenol-chloroform method.

#### PCR amplification

The primers CSX1XR (ATTTTTCGCGATTTTCGCAGAACG) and CSX2XR (CGAGTAGCAGAAACTCCCGTTCA), and 13Z (ACTGGGGGTTGGTGTAAAATAG) and LiR (TCGCAGAACGCCCCT) as external and internal pair primers were employed to amplify intended part of *kDNA*, respectively [21]. Accordingly, these primers amplify about 570 bp, 750 bp, and 720 bp for *L*. *major*, *L*. *tropica*, and *L*. *infantum*. The thermocycler program was set for 30 cycles for the first stage of amplification. The conditions of this stage were as follows: an initial denaturation for 5 minutes at 94°C, denaturation for 1 minute at 94°C, anneal for 1 minute at 55°C, extension for 1 minute at 72°C, and a final extension phase of 5 minutes at 72°C. The annealing temperature for the second stage was 57 degrees Celsius, which was the sole variation between the first and second phases. On 1.5% concentration agarose gels, the amplification results were analyzed. The pooling technique was utilized to save costs and time for sandfly molecular study, and the specimens of positive pools were examined separately.

#### Sequencing and phylogenetic analysis

The amplicons of the *kDNA* gene of six isolated *L*. *infantum* were directly sequenced (Bioneer Korea and Codon Genetics) to identify *Leishmania* species accurately. The multiple sequences were edited and compared to RefSeq (Accession number: KU587709) using the Sequencher software (Tmv.4.1.4). The multiple sequence alignment was carried out by BioEdit software based on ClustalW method. A phylogenetic tree was produced by MEGA software (version 5.05) based on the Maximum Likelihood technique and Kimura 2-parameter model to authenticate phylogeny relationships among the *Leishmania* spp. The calculated distance scale was 0.05. *Trypanosoma congolense* (accession number: M19750) was considered an out-group branch. The bootstrap values of higher than 60% supported the topology on each branch. Based on the analysis of molecular variance (AMOVA), the number of haplotypes (Hn), genetic (Haplotype) diversity (Hd), and nucleotide diversity (Nd) were calculated using DnaSP software (version 5.10).

## Results

### Molecular detection and species identification of *Leishmania* parasite

**Fig 1:**
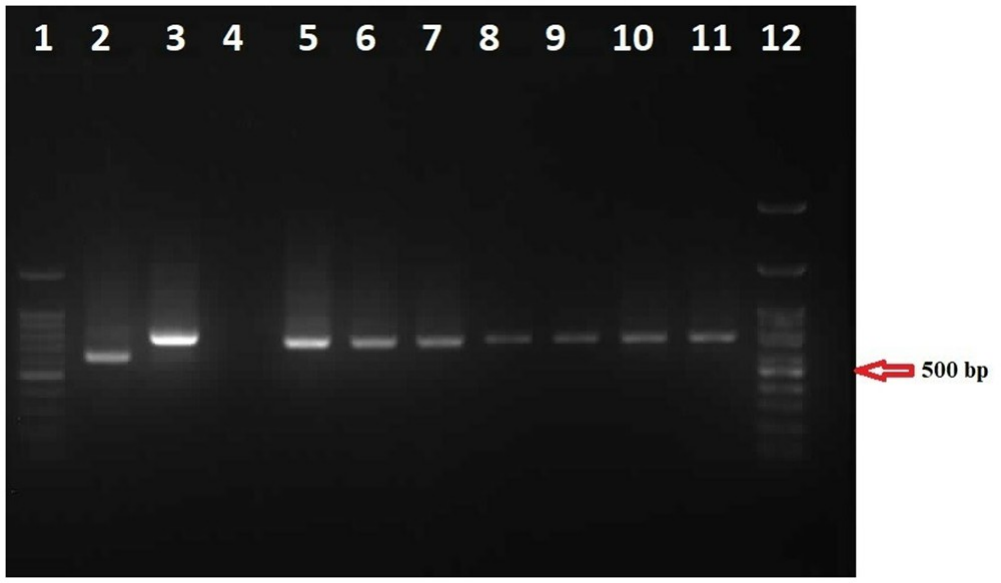
An agarose gel of amplified kDNA. The gel contained two 100 bp markers to estimate amplified fragments size, three positive controls, a negative control, and six human, dog, and sandfly samples. Lanes 1 and 12: marker (100 bp, sinaclon, Iran), Lane 2: *L*. *major* (MHOM/IR/75/ER) about 560 bp length, Lanes 3: *L*. *tropica* (MRHO/IR/75/ER) about 750 bp length, Lane 4: negative control, Lane 5: *L*. *infantum* (MCAN/IR/07/Moheb.gh) about 720 bp length, Lanes: 6–8: human samples about 720 bp length, Lane 9: sandfly samples, Lanes 10 and 11: dog samples. All samples of the present study (lanes 6 to 11) illustrated a product size of ≈720 bp.

As mentioned, all seropositive human and dog samples, 10 percent of seronegative human samples, and all female *Phlebotomus* spp were subjected to molecular investigation. The specific kinetoplast minicircle DNA with a length of 720 bp for *L*. *infantum* was amplified using Nested-PCR in thirty samples, including 20 human samples, eight dog samples, and two sandfly samples (Fig 1).

Regarding human samples, among all individuals with anti-*L*. *infantum* antibodies at titers above 1:800 (31 cases), *kDNA* of *L*. *infantum* was detected in 20 samples. *Leishmania* parasite was detected in all human samples with a titer ≥1:3200 (all ten available seropositive samples). Ten other molecular positive samples were among 18 human blood samples with titer 1:800 to 1:1600. All seronegative samples were found also negative in molecular investigations.

We have access to the blood sample of 8 out of ten seropositive dogs (titer above 1:320). Interestingly, the *kDNA* of *L*. *infantum* was detected in all seropositive ones.

Regarding Phlebotominae sandflies, *L*. *infantum* was detected in two pools, each containing five female specimens of *Major group*; based on total examined female specimens (277), the minimum infection rate was calculated as 2.98%. Individuals from each positive pool were investigated independently to determine the infection rate, and it was discovered that each pool included only one contaminated sample. Thus, a 0.72 percent infection rate was determined. Studying the abdominal condition of infected sandflies revealed that the abdomens of both samples were empty (without blood).

### Phylogenetic analysis

**Fig 2:**
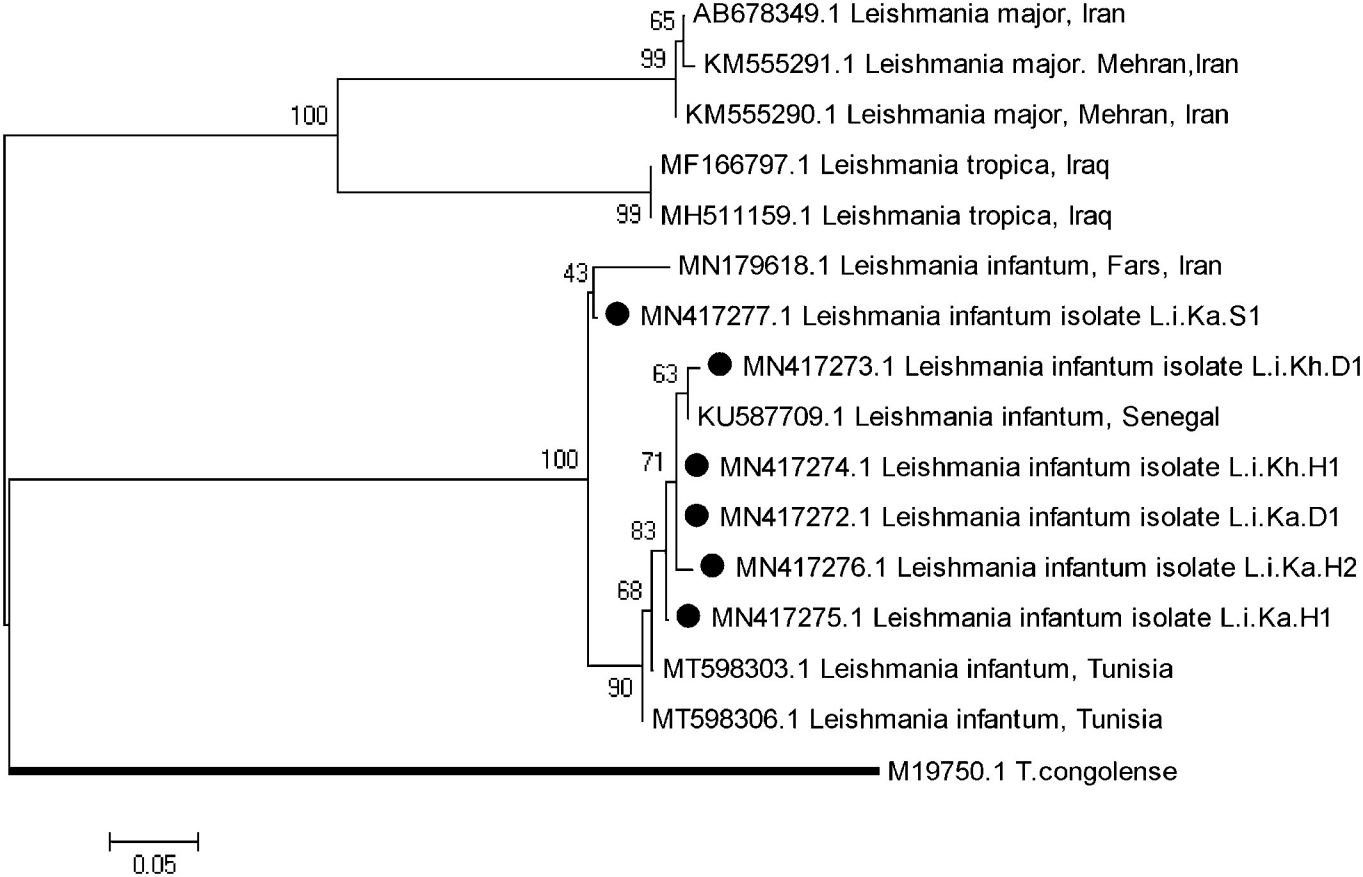
A Maximum-likelihood phylogenetic tree generated for a total of 440 bp of the *kDNA* gene fragments concatenated from a few members of the genus *Leishmania* obtained from humans, dogs and sandflies in Kaleybar and Khoa-Afarin counties, East Azerbaijan, Iran (characterized by circular filled shape), and already available on the GenBank database. Also, *Trypanosoma congolense* (accession number: M19750) was considered as an out-group branch. The bootstrap values of higher than 60% supported the topology on each branch.

**Fig 3:**
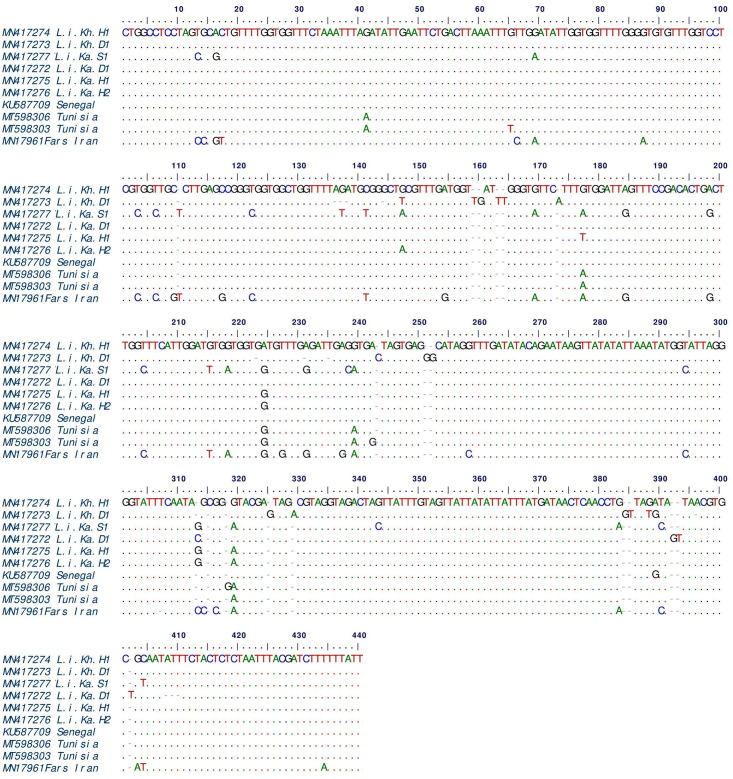
Multiple sequence alignment compared the nucleotides of *L*. *infantum kDNA* gene traced from human (L.i.Kh.H1: MN417274, L.i.Ka.H1: MN417275 and L.i.Ka.H2: MN417276), sandfly (L.i.Ka.S1: MN417277) and dog (L.i.Ka.D1: MN417272 and L.i.Kh.D1: MN417273) in the present study.

A total of 30 *Leishmania*-positive PCR products from the human, dog, and mosquito samples were sent to be sequenced in two steps. Unfortunately, only the sequences of 6 parasites were discovered to be appropriate for examination and deposition in the GenBank among the sequenced samples, even though they were repeated (Accession NOs: MN417272 to MN417277), the approximate sampling location of the sequenced parasites is shown on the region map in S1 Fig. The sequenced samples include three *Leishmania* infantum sequences related to humans (Accession NOs: MN417274, MN417275, and MN417276), two from dogs (Accession NO: MN417272 and MN417273), and one parasite from sandflies (Accession NO: MN417277) (Table 1). For the authentication of the taxonomic status of *Leishmania* sp., a phylogenetic tree was generated based on allelic differentiation. The topology of identified parasites indicated that all detected *Leishmania* parasites belonged to *L*. *infantum*, which was placed at their specific clade (Fig 2). The critical finding is that there are considerable differences between the parasite sequences found in sandflies and those found in dogs and humans. The two samples (Li.Ka.H1 and Li.Ka.H2) connected to the parasite found in two persons from the Kaleybar area have the most significant similarity (99.6%). Based on multiple nucleotide alignment, the synonymous substitutions of *L*. *infantum* isolated from human (L.i.Ka.H), sandfly (L.i.Ka.S), and dog (L.i.Ka.D) were observed in different positions (Fig 3). The heterogeneity analysis of *kDNA* sequences of *L*. *infantum* showed a significant genetic diversity (Hd; 1, Hn: 6). However, the nucleotide differences showed a low diversity (Nd; 0.00926).

**Table 1:** Source of parasites DNAs, DAT result and collection site of GenBank submitted sequences.

GenBank Accession No.	Source	DAT results	Sample site
MN417272	Dog	Positive	Kaleybar
MN417273	Dog	Positive	Khoda-Afarin
MN417274	Human	Positive	Khoda-Afarin
MN417275	Human	Positive	Kaleybar
MN417276	Human	Positive	Kaleybar
MN417277	Sand-fly	-	Kaleybar

## Discussion

The study area is considered one of the most prominent endemic foci of VL in Iran, and 13 cases were officially reported during the last five years. On the other hand, no study was carried out during the last two decades on human and animal reservoirs. Therefore, conducting an epidemiological study and clarifying the various aspects of VL in the region seemed necessary. Following our previous study, serological and microscopical parts, the necessity of molecular investigation to reveal other aspects of the disease in the region was realized.

The selection of an appropriate molecular marker was essential to commence the study; there are numerous options for these purposes, such as *ITS*, *CytB*, and *kDNA*; each has advantages and disadvantages. The genetic marker should be chosen based on the purpose of the study and available samples; even though the first two mentioned markers are more suitable tools for studying genetic diversity [22, 23], the significant privilege of *kDNA* is its high copy number and detectability in samples with low parasite loads [24], so, *kDNA* was targeted to improve the detection limit. *Leishmania* DNA was detected in samples with various titers. For instance, based on the DAT test, human sera with titers 1:800 and 1:1600 are regarded as negative and suspicious, respectively, but the intended fragment was amplified in those samples using PCR. Serology and molecular results revealed high agreements so that DNA of the parasite was detected in 53.33%, 66.36%, and 100% of available human blood samples with titer 1:800, 1:1600, and ≥ 3200, respectively. Moreover, the desired DNA fragment was amplified in all dog blood samples with titer ≥ 320. On the other hand, any seronegative or samples with lower than mentioned titers were not PCR- positive. The critical point is that the percentage of positive samples increases with higher titers, suggesting a correlation between titer levels and the likelihood of detecting the parasite’s DNA. These findings emphasize the importance of using serological and molecular techniques for accurate diagnosis.

Species determination was another goal of the present study, and the selected marker can identify the species of the parasite based on the size of the resulting band. The size of amplified bands of the *kDNA* in all samples, including humans, dogs, and sandflies, was 720 bp, which coordinates with the expected size for *L*. *infantum*. This result aligns with earlier studies conducted in nearby and other Mediterranean areas [25–27]. Detecting *L*. *infantum* parasites in various samples (humans, dogs, and sandflies) in Kaleybar and Khoda-Afarin Counties brings up several important points; 1) It suggests that visceral leishmaniasis caused by *L*. *infantum* is a significant health concern, highlighting the need for targeted public health interventions to reduce the disease burden, 2) the tracing of DNA of *L*. *infantum* in domestic dog samples confirms their role as reservoir hosts and contributes to the parasite’s spread. It is important to note that other studies have also claimed this issue [28]. Therefore, measures to screen, prevent, and treat infected dogs are crucial to minimize the risk of transmission to humans. A previous study in the same region has previously corroborated this issue [29]. Applying insecticide-treated collars on domestic canines in that study led to a significant decline in disease incidents within the examined villages, 3) The detection of *L*. *infantum* in sandfly samples indicates their role as vectors in transmitting the parasite. Understanding local sandfly species’ distribution and behavior is essential for implementing effective vector control measures, 4) Using molecular techniques for accurately identifying *Leishmania* species, such as PCR analysis, is essential for designing appropriate control strategies. The finding that *L*. *infantum* is the primary species detected suggests that this strain is the main driver of leishmaniasis transmission in the study region.

In the case of Phlebotominae sandflies, *Leishmania* infection was detected in two specimens belonging to Major group. As a drawback, the female member of this group (*P*. *perfiliewi*, *P*. *tobbi*, *P*. *neglectus* and/or *P*. *major* s.str) cannot be identified at the species level based on the common morphological characters [30]. One suggested way to approximate the species of female specimens of Major group is to determine the composition of male samples in the same sampling set. Based on a previously published study by the same authors, the dominant species was *P*. *perfiliewi* among three existing species in the study area (*P*. *neglectus*, *P*. *tobbi*, and *P*. *perfiliewi*), so it can be assumed that the infected species were perhaps *P*. *perfiliewi* [19], which its role in VL transmission was also highlighted by other researchers in Northwest Iran [20]. The presence of *Leishmania* infection in these specimens raises essential questions about the prevalence and spread of this parasite in the Kaleybar and Khoda-Afarin Counties. *Leishmania* is a parasitic vector-borne disease that affects both humans and animals, causing a range of clinical manifestations. Therefore, understanding the species of sandflies that are carriers of *Leishmania* is crucial for effective disease control and prevention strategies. It is worth noting that identifying the infected species as *P*. *perfiliewi* is an assumption based on the dominant species observed in the previous study. While this assumption is reasonable, further research is needed to confirm the identity of the infected sandflies. More specifically, molecular techniques such as DNA sequencing or PCR could be employed to definitively determine whether *P*. *perfiliewi* was indeed the infected species in these specimens. Furthermore, this finding highlights the need for ongoing *Leishmania* infection surveillance and monitoring of sandflies in Kaleybar and Khoda-Afarin Counties. Continued research efforts will help to shed light on the dynamics of *Leishmania* transmission in this area, leading to improved prevention and control strategies.

The detection of parasites in sandflies with empty abdomens indicates at least one-time previous blood feeding. The isolation of parasites at this stage of the blood-feeding cycle of sandflies is essential from an epidemiological point of view. Based on molecular methods, confirming the presence of the parasite’s metacyclic stage in the sandfly’s midgut is impossible. However, seeing the parasite after blood digestion is helpful since it demonstrates that it has finished the phases of blood feeding and, more crucially, has removed the sandfly’s peritrophic membrane and is still present in its digestive system. It is, therefore, a relatively strong guarantee of the possibility of sandfly’s role in disease transmission [31].

As mentioned, the genetic diversity analysis was performed on the six sequences. Haplotype diversity was high in the current study (Hd:1); this index estimates the diversity of DNA sequences and scored from Zero to 1 so that our result indicates the highest level of diversity. This finding suggests that the isolates vary significantly from each other at the genetic level, indicating a broad range of distinct genotypes within the *L*. *infantum* population under investigation.

Two *Leishmania* DNAs that were recovered from blood samples of patients in Kaleybar County had the greatest degree of sequence similarity, which may be explained by the same geographic area. On the other hand, the lowest similarity was detected among the sequences of DNAs that were extracted from a sandfly captured from Kaleyber County and a dog from Khoda-Afarin County. This issue can be in terms of the difference in the hosts and may be due to the different sampling regions.

High genetic diversity is a typical characteristic of *kDNA*, as pointed out by other researchers. Cortes et al. reported a high degree of polymorphism in the *kDNA* gene of *L*. *infantum* isolates, and Kariyawasam et al. reported high haplotype diversity (0.757) in *L*. *donovani* in Portugal and Sri Lanka [32, 33]. This character was found in other *Leishmania* species [34].

Despite the inherent diversity of *kDNA*, the genetic diversity of the present study sample may have several implications. Firstly, it suggests that *L*. *infantum* exhibits a high degree of genetic variability, which could have significant implications for understanding its epidemiology and transmission dynamics. Genetic diversity within a population can influence virulence, drug resistance, and host specificity. Therefore, a high level of diversity necessitates additional investigations to determine its potential implications regarding disease severity, treatment strategies, and public health. Furthermore, the high haplotype diversity may have important implications for developing effective control and prevention measures against *L*. *infantum*. The diverse genotypes suggest that this pathogen can adapt to different environmental conditions, hosts, and vectors. This adaptability could result in the emergence of new strains or the spread of existing ones, complicating efforts to control and eliminate the disease.

To sum up, the results approved that VL is a significant health problem in the study region and should be consistently monitored by the district health office. The present study unearthed that about 10% of domestic dogs were infected by *L*. *infantum* in Kaleybar and Khoda-Afarin counties, so the disease should be controlled in dogs to interrupt the parasite circulation. Therefore, *kDNA* seems a suitable marker for *Leishmania* detection in the samples with low amounts of parasite, such as in blood samples and sandflies. Therefore, DAT and *kDNA* nested PCR may be considered competent methods for detecting the disease in humans and dogs. *L*. *infantum* is the main agent of VL in northwestern part of Iran and showed a high genetic diversity based on *kDNA*. Furthermore, a correlation was observed among parasites’s genetic heterogeneity, geographic origin, and the host.

### Limitations

The study was limited by the inaccessibility of the region and some dog owners’ reluctance to participate, which hindered the ability to collect more blood samples from dogs.

## Supporting information

S1 Fig The uncropped and original agarose gel of amplified kDNA samples of the present study beside three control positive, a control negative, and two 100 bp markers. (PDF)

S1 Table The geographical coordinates of the villages from which the sequenced samples were taken. (DOCX)

## References

[1] Alvar J, Vélez ID, Bern C, Herrero M, Desjeux P, Cano J, et al. Leishmaniasis worldwide and global estimates of its incidence. PloS one. 2012;7(5):e35671. doi: 10.1371/journal.pone.0035671 22693548 PMC3365071

[2] Mohebali M. Visceral leishmaniasis in Iran: review of the epidemiological and clinical features. Iranian journal of parasitology. 2013;8(3):348. 24454426 PMC3887234

[3] Sabzevari S, Teshnizi SH, Shokri A, Bahrami F, Kouhestani F. Cutaneous leishmaniasis in Iran: A systematic review and meta-analysis. Microbial pathogenesis. 2021:104721. doi: 10.1016/j.micpath.2020.104721 33539962

[4] Organization WH. Global leishmaniasis surveillance: 2019–2020, a baseline for the 2030 roadmap. 2021.

[5] Alvar J, Yactayo S, Bern C. Leishmaniasis and poverty. Trends in parasitology. 2006;22(12):552–7. doi: 10.1016/j.pt.2006.09.004 17023215

[6] Gradoni L. The Leishmaniases of the Mediterranean region. Current Tropical Medicine Reports. 2017;4(1):21–6. 10.1007/s40475-017-0101-y PMC5362652 28386524

[7] Sundar S, Singh OP. Molecular diagnosis of visceral leishmaniasis. Molecular diagnosis & therapy. 2018;22(4):443–57. 29922885 10.1007/s40291-018-0343-y PMC6301112

[8] Srividya G, Kulshrestha A, Singh R, Salotra P. Diagnosis of visceral leishmaniasis: developments over the last decade. Parasitology research. 2012;110(3):1065–78. doi: 10.1007/s00436-011-2680-1 22065060

[9] Mohebali M, EDRISIAN GH, Nadim A, Hajaran H, Akhoundi B, HOUSHMAND B, et al. Application of direct agglutination test (DAT) for the diagnosis and seroepidemiological studies of visceral leishmaniasis in Iran. 2006.

[10] Mohebali M, Keshavarz H, Shirmohammad S, Akhoundi B, Borjian A, Hassanpour G, et al. The diagnostic accuracy of direct agglutination test for serodiagnosis of human visceral leishmaniasis: a systematic review with meta-analysis. BMC Infectious Diseases. 2020;20:1–12. doi: 10.1186/s12879-020-05558-7 33308170 PMC7729288

[11] Echchakery M, Chicharro C, Boussaa S, Nieto J, Ortega S, Carrillo E, et al. Molecular identification of Leishmania tropica and L. infantum isolated from cutaneous human leishmaniasis samples in central Morocco. 2020. 10.4103/0972-9062.308804 33818459

[12] Ortuño M, Latrofa MS, Iborra MA, Pérez-Cutillas P, Bernal LJ, Risueño J, et al. Genetic diversity and phylogenetic relationships between Leishmania infantum from dogs, humans and wildlife in south‐east Spain. Zoonoses and public health. 2019;66(8):961–73. doi: 10.1111/zph.12646 31512370

[13] Marcelino AP, de Souza Filho JA, e Bastos CdV, Ribeiro SR, Medeiros FAC, Reis IA, et al. Comparative PCR-based diagnosis for the detection of Leishmania infantum in naturally infected dogs. Acta tropica. 2020;207:105495. doi: 10.1016/j.actatropica.2020.105495 32305295

[14] Flaih MH, Al-Abady FA, Hussein KR. Phylogenetic analysis of kinetoplast DNA: kDNA of Leishmania tropica in Thi-Qar province, Iraq. Comparative immunology, microbiology and infectious diseases. 2021;78:101696. doi: 10.1016/j.cimid.2021.101696 34416483

[15] Mohammadpour I, Hatam GR, Handjani F, Bozorg-Ghalati F, PourKamal D, Motazedian MH. Leishmania cytochrome b gene sequence polymorphisms in southern Iran: relationships with different cutaneous clinical manifestations. BMC infectious diseases. 2019;19(1):1–13. 30696426 10.1186/s12879-018-3667-7 PMC6352432

[16] Samei N, Khatami Nezhad M, Yaghoubinezhad A, Najafzadeh N, Spotin A, editors. Finding various molecular haplotypes of Leishmania major in human using three HSp70, ITS-rDNA and Cyt b genes. Proceedings of 1 st and 13 th Iranian Genetics Congress Tehran Iran; 2014.

[17] Najafzadeh N, Taslimian R, Fotouhi-Ardakani R, Spotin A, Parvizi P. Detection and differentiation of Leishmania parasites in asymptomatic canine by High-Resolution Melting analysis of microsatellite fragment in ITS gene. Microbial Pathogenesis. 2022;162:105300. doi: 10.1016/j.micpath.2021.105300 34808275

[18] Aghamolaei S, Behniafar H, Behravan M, Hajjaran H, Moin Vaziri V. Probability of false-negative results in microscopical detection of cutaneous leishmaniasis: more accurate screening by kDNA-PCR during epidemiological survey. Journal of Parasitic Diseases. 2020;44(4):781–4. doi: 10.1007/s12639-020-01246-0 33184545 PMC7596115

[19] Behniafar H, Moin-Vaziri V, Mohebali M, Tabaei SJS, Zarei Z, Kazemirad E, et al. Visceral leishmaniasis among children in an endemic area of northwestern Iran between 2016 and 2017: An epidemiological study. Asian Pacific Journal of Tropical Medicine. 2019;12(7):306.

[20] Parvizi P, Mazloumi-Gavgani A, Davies C, Courtenay O, Ready P. Two Leishmania species circulating in the Kaleybar focus of infantile visceral leishmaniasis, northwest Iran: implications for deltamethrin dog collar intervention. Transactions of the Royal Society of Tropical Medicine and Hygiene. 2008;102(9):891–7. doi: 10.1016/j.trstmh.2008.04.026 18554675

[21] Noyes HA, Reyburn H, Bailey JW, Smith D. A nested-PCR-based schizodeme method for identifying Leishmania kinetoplast minicircle classes directly from clinical samples and its application to the study of the epidemiology of Leishmania tropica in Pakistan. Journal of clinical microbiology. 1998;36(10):2877–81. doi: 10.1128/JCM.36.10.2877-2881.1998 9738037 PMC105081

[22] Ghatee MA, Mirhendi H, Karamian M, Taylor WR, Sharifi I, Hosseinzadeh M, et al. Population structures of Leishmania infantum and Leishmania tropica the causative agents of kala-azar in Southwest Iran. Parasitology research. 2018;117(11):3447–58. doi: 10.1007/s00436-018-6041-1 30105405

[23] Benikhlef R, Chaouch M, Ben Abid M, Aoun K, Harrat Z, Bouratbine A, et al. Its1 and Cpb Genetic Polymorphism of Algerian and Tunisian Leishmania Infantum Isolates from Humans and Dogs. Leishmania Infantum. 10.1111/zph.13016 36443904

[24] Behniafar H, Vaziri VM, Tabaei SJS, Taghipour N. Comparison of Three Commonly Used Genetic Markers for Detection of Leishmania Major: An Experimental Study. Ethiopian Journal of Health Sciences. 2021;31(4). doi: 10.4314/ejhs.v31i4.6 34703171 PMC8512936

[25] Rostamian M, Bashiri H, Yousefinejad V, Bozorgomid A, Sohrabi N, Raeghi S, et al. Prevalence of human visceral leishmaniasis in Iran: A systematic review and meta-analysis. Comparative immunology, microbiology and infectious diseases. 2021;75:101604. doi: 10.1016/j.cimid.2020.101604 33388595

[26] Soleimani A, Mohebali M, Gholizadeh S, Bozorgomid A, Shafiei R, Raeghi S. Molecular and serological evaluation of visceral leishmaniasis in domestic dogs and cats in Maragheh County, north-west of Iran, 2018–2021. Veterinary Medicine and Science. 2022. doi: 10.1002/vms3.846 35622829 PMC9514466

[27] Tabatabaie F, Nasirikaleybar Y, Mohebali M, Solgi R, Babaei V, Heidari Z, et al. Serological and molecular survey of zoonotic visceral leishmaniasis in stray dogs (Canis familiaris) from an endemic focus in Meshkin-Shahr district in Ardabil province, Iran. Journal of Vector Borne Diseases. 2021;58(3):213. doi: 10.4103/0972-9062.325636 35170458

[28] Gavgani A, Mohite H, Edrissian GH, Mohebali M, Davies CR. Domestic dog ownership in Iran is a risk factor for human infection with Leishmania infantum. The American journal of tropical medicine and hygiene. 2002;67(5):511–5. doi: 10.4269/ajtmh.2002.67.511 12479553

[29] Courtenay O, Bazmani A, Parvizi P, Ready PD, Cameron MM. Insecticide–impregnated dog collars reduce infantile clinical visceral leishmaniasis under operational conditions in NW Iran: a community–wide cluster randomised trial. PLoS neglected tropical diseases. 2019;13(3):e0007193. doi: 10.1371/journal.pntd.0007193 30830929 PMC6417739

[30] Nadim A, Javadian E. Key for species identification of sandflies (Phlebotominae; Diptera) of Iran. 1976.

[31] Kamhawi S. Phlebotomine sandflies and Leishmania parasites: friends or foes? Trends in parasitology. 2006;22(9):439–45. doi: 10.1016/j.pt.2006.06.012 16843727

[32] Cortes S, Mauricio I, Almeida A, Cristovão JM, Pratlong F, Dedet JP, et al. Application of kDNA as a molecular marker to analyse Leishmania infantum diversity in Portugal. Parasitology international. 2006;55(4):277–83. doi: 10.1016/j.parint.2006.07.003 16959531

[33] Kariyawasam UL, Selvapandiyan A, Rai K, Wani TH, Ahuja K, Beg MA, et al. Genetic diversity of Leishmania donovani that causes cutaneous leishmaniasis in Sri Lanka: a cross sectional study with regional comparisons. BMC Infectious Diseases. 2017;17(1):1–11. 29273010 10.1186/s12879-017-2883-x PMC5741890

[34] Oryan A, Shirian S, Tabandeh M-R, Hatam G-R, Randau G, Daneshbod Y. Genetic diversity of Leishmania major strains isolated from different clinical forms of cutaneous leishmaniasis in southern Iran based on minicircle kDNA. Infection, Genetics and Evolution. 2013;19:226–31. doi: 10.1016/j.meegid.2013.07.021 23892374

