# Supplementary figures and images for: Detection and phylogenetic analysis of kinetoplast DNA of Leishmania infantum infected humans, domestic dogs and sandflies in Northwest Iran
Source: PLoS One. 2024 Mar 13;19(3):e0296777. doi: 10.1371/journal.pone.0296777 (PMC10936802; doi:10.1371/journal.pone.0296777)

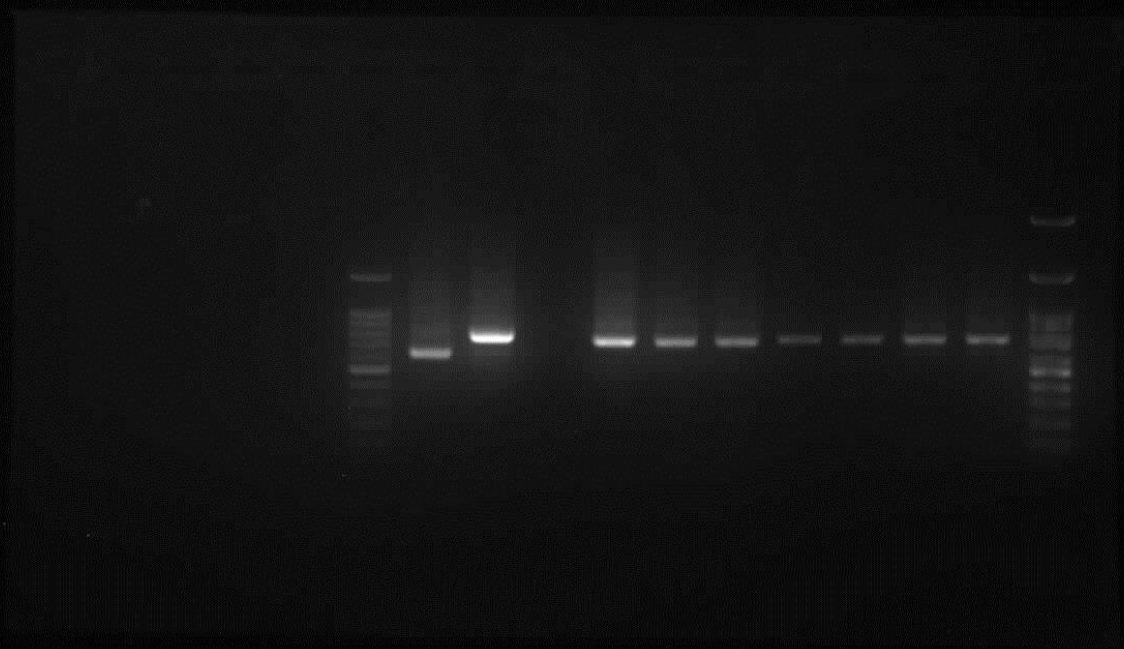

Supplement: S1 Fig — (PDF) [file pone.0296777.s001.pdf]
