# Supplementary material for: Detection and phylogenetic analysis of kinetoplast DNA of Leishmania infantum infected humans, domestic dogs and sandflies in Northwest Iran
Source: PLoS One. 2024 Mar 13;19(3):e0296777. doi: 10.1371/journal.pone.0296777 (PMC10936802; doi:10.1371/journal.pone.0296777)
Supplement: S1 Table — (DOCX) [file pone.0296777.s002.docx]

| Accession number | Parasite isolated village (County) | Latitude | Longitude |
| --- | --- | --- | --- |
| MN417272 | Najaf-Tatakameh (Kaleybar) | 39.082926 N | 47.3992451 E |
| MN417273 | Gheshlaq-Uzbak (Khoda-Afarin) | 39.228853 N | 47.294024 E |
| MN417274 | Aliverdi Ushaqi (Khoda-Afarin) | 39.228187 N | 47.152046 E |
| MN417275 | Ghayeh bashi (Kaleybar) | 39.109674 N | 47.218620 E |
| MN417276 | Delavaran OR Dilbilmaz (Kaleybar) | 39.105437 N | 47.284856 E |
| MN417277 | Kalalaq (Kaleybar) | 38.824000 N | 47.065037 E |
